# Supplementary material for: Oral Microbiome: A Review of Its Impact on Oral and Systemic Health
Source: Microorganisms. 2024 Aug 29;12(9):1797. doi: 10.3390/microorganisms12091797 (PMC11434369; doi:10.3390/microorganisms12091797)
Supplement: Supplementary file 1 [file microorganisms-12-01797-s001.zip › microorganisms-3110227-supplementary.pdf]

Oral Microbiome: A Comprehensive Review of Its Impact on Oral and Systemic Health

John J. Rajasekaran [1,*], Hari Krishnan Krishnamurthy [1], Jophi Bosco [2], Vasanth Jayaraman [1], Karthik Krishna [1], Tianhao Wang [1] and Kang Bei [1]

[1] Vibrant Sciences LLC, Santa Clara, CA 95054, USA; hari@vibrantsci.com (H.K.K.); vasanth.jayaraman@vibrantsci.com (V.J.); karthik@vibrantsci.com (K.K.); tianhao.wang@vibrantsci.com (T.W.); kang@vibrantsci.com (K.B.)

[2] Vibrant America LLC, Santa Clara, CA 95054, USA; jophi.b@vitasoft-tech.com

* Correspondence: jjrajasekaran@vibrantsci.com; Tel.: +1-5094325707

Oral Microbiome and Techniques for Analysis

Researchers employ several techniques to analyse the oral microbiome, with culture-dependent and culture-independent methods offering distinct advantages and limitations. Culture-dependent methods have been conventionally used. The advantage of using these methods is that microorganisms may be identified and described according to their shape, growth patterns, and biochemical characteristics. In the past, these techniques have offered insightful knowledge on the physiology and behaviour of particular cultivable species. They can be particularly useful in identifying potential pathogens and studying their responses to different conditions. However, culture-dependent techniques have inherent biases, favouring the growth of easily culturable species while missing a substantial portion of the microbial community that is difficult to grow in the lab [1]. It is difficult to cultivate many of the fastidious or anaerobic microorganisms found in the oral microbiome [11]. Furthermore, the microbial makeup may change during the cultivation process, producing an imprecise depiction of the actual oral microbiome.

On the other hand, next-generation sequencing (NGS) methods, which are microbial analysis techniques independent of culture, have led to a notable advancement in the research of the oral microbiome. These advanced tools have ushered in new possibilities for conducting extensive metagenomic studies across diverse populations [12]. This has led to the detailed characterization of the microbiome's structural composition and, in certain cases, the elucidation of its functional roles and implications for human health [13]. As the costs associated with these techniques continue to decrease, both in terms of financial investment and computational requirements, researchers are empowered to harness their potential. This trend aligns with the ongoing expansion of microbial genetic sequence databases, further enhancing the capabilities of culture-independent NGS methods. Consequently, these techniques not only expedite analyses but also significantly amplify our understanding of previously challenging-to-study, unculturable, and rare microbiota [13]. Culture-independent methods, particularly those involving DNA or RNA

analysis extracted directly from samples, represent a pivotal transformation in comprehending the oral microbiome. Techniques such as 16S rRNA gene sequencing and shotgun metagenomics are commonly used tools for identification and offer distinct advantages by providing an impartial perspective of the oral microbiome. This comprehensive approach enables the identification of elusive microbial species that could potentially hold significant roles in oral health and disease dynamics [1].

Nonetheless, culture-independent methods do have their limitations. Although they excel at indicating microbial species, they cannot often furnish insights into the specific physiological characteristics and functional behaviours of individual organisms. Additionally, their increased sensitivity may result in the detection of DNA from non-viable or deceased cells, potentially inflating assessments of actual microbial activity. The emergence of NGS, a prominent subset of culture-independent methods, has revolutionised the landscape of oral microbiome analysis. NGS techniques, exemplified by Illumina sequencing, stand out for their capacity to analyse a substantial number of samples in a high-throughput manner [14,15]. The data generated through NGS is both expansive and rapid, making it a cost-effective tool. NGS explores the functional potential of the oral microbiome in addition to capturing its taxonomic diversity. Consequently, NGS technologies have greatly expanded our knowledge of previously difficult-to-studied microbial species, leading to a more thorough understanding of the oral environment.

Nevertheless, RT-PCR is still the recommended technique for identifying oral cavity microorganisms, even with the benefits of NGS. RT-PCR is still the preferred technique for identifying microorganisms in the oral cavity, even with the advantages that NGS offers. This decision is explained by its inexpensiveness, speed, specificity, and low sample requirements in comparison to NGS, which requires more time and resources [16]. Although RT-PCR is frequently used by academics for regular analysis, NGS is usually chosen for in-depth studies of microbial communities. The remarkable sensitivity of RT-PCR enables the prompt and accurate identification of oral pathogens linked to a range of oral disorders, such as dental caries, periodontal diseases, and oral candidiasis. Its heightened sensitivity enhances precision in quantification and identification, making it invaluable for accurate microbial analysis [17]. This method provides cost-saving benefits by reducing the need for extensive post-PCR detection procedures. Additionally, they enable the simultaneous identification of multiple pathogens within a single sample, enhancing efficiency in diagnostic and research applications [18]. Other advantages include ease of quantification, reproducibility, quality control, and reduced risk of contamination [19].

The utilisation of stimulated saliva collection has emerged as a favoured approach for investigating the intricacies of the oral microbiome. By introducing specific stimuli

like chewing gum or citric acid, stimulated saliva is acquired under controlled and standardised conditions, setting it apart from unstimulated saliva. This methodical stimulation ensures a consistent and reproducible sampling process, effectively minimising variations arising from factors such as circadian rhythms. Furthermore, the elevated microbial load and diversity found in stimulated saliva yield a more comprehensive representation of the oral microbiome, thereby facilitating a more insightful analysis of its composition and functions [20,21]. Moreover, saliva's significance extends beyond its role in the oral microbiome. It envelops the entire oral cavity, consistently introducing its bacterial constituents into other oral samples. In essence, saliva serves as an illustrative example of a microbiome characterised by high alpha diversity but low beta diversity [22]. This unique combination of attributes renders stimulated saliva collection a potent tool for comprehending the complex interaction between microbial communities within the oral ecosystem, paving the way for enhanced insights into its implications for human health.

Practices for Daily Dental Care

Moderate Tea and Coffee Consumption: Extrinsic tooth pigmentation can also be caused by tea and coffee, two popular drinks. The dark pigments that are present in them have the potential to progressively discolour enamel, resulting in obvious discoloration over time. These drinks, along with tobacco, red wines, and colas, are known to leave surface stains on teeth. Given that tea and coffee can discolour teeth, dentists highly encourage patients to limit their consumption of these beverages [267]. In addition to reducing the chance of discoloration, rinsing with water after consumption can help maintain the teeth's natural brightness.

Bedtime oral care routine: Implementing a nighttime oral hygiene regimen is important for preserving dental health. To eliminate food particles and plaque accumulation, this regimen should include brushing with fluoride toothpaste, particularly before bed [268] Tooth decay can also be avoided by abstaining from sugar-filled meals and beverages right before bed [268]. Maintaining this evening practice consistently lowers the chance of dental problems and improves oral hygiene.

Twice-Daily Brushing: Although some people think that brushing once a day is enough, most dentists advise brushing twice a day in order to improve plaque control and preserve dental health. Numerous research has demonstrated the usefulness of this approach, which is commonly adopted by patients. While there is conflicting research regarding the best time to brush—before or after meals—post-meal brushing may help prevent food impaction and lessen the effects of sugar on teeth [269].

Gentle Brushing Techniques: Brushing with a soft-bristled toothbrush for two to three minutes twice a day is considered gentle brushing. The Bass technique, which involves sweeping or circular strokes with minimal force, is an excellent way to eliminate plaque without harming teeth or gums. By avoiding gum irritation and enamel deterioration, these procedures encourage the best possible dental hygiene [270].

Daily Flossing: The American Academy of Periodontology suggests incorporating daily flossing into a regular oral care regimen to diminish the occurrence and intensity of gingival inflammation linked to periodontal ailments [271]. Flossing at least once a day is essential for removing plaque and debris from between teeth and along the gumline, where toothbrushes cannot reach effectively.

Water Picks as an Alternative: By using a power-driven stream of water to remove plaque and debris from in between teeth and beneath the gum line, water picks offer a milder alternative to traditional flossing. Compared to ordinary floss and interdental brushes, it has greater results on dental plaque removal and gingivitis control because it can transport antimicrobial solutions into the sulcus and interproximal regions. In orthodontic patients, it has also demonstrated effectiveness in treating gingivitis and peri-implant mucositis. According to studies, using water flossers every day for up to three months is safe and does not raise the risk of bacteremia while receiving periodontal maintenance therapy [272].

Tongue Cleaning: By eliminating tongue coating, mechanical tongue cleaning—which includes techniques like tongue scraping—has been found to be an efficient way to lessen foul breath [273]. Research indicates that using tongue scrapers instead of toothbrushes can effectively reduce bad breath, emphasizing the need of including tongue cleaning in oral hygiene practices [274].

Safety of Sealants: The use of pit and fissure sealants has been proven to be a safe and effective preventive measure in reducing and preventing dental caries, particularly on the occlusal surfaces of molars. Studies have demonstrated significant reductions in caries incidence among children treated with sealants compared to untreated control groups, highlighting the importance of incorporating sealants into preventive dental care practices [275].
